# A Practical Treatise on the Diseases of Children

**Published:** 1842-01

**Authors:** 


Art. XI.—*A Practical Treatise on the Diseases of Children.* By James Stewart, m.d.—*New York,* 1841. 8vo, pp. 544.

We have been disappointed in Dr. Stewart's book. It is professedly a compilation—and it is obviously a compilation. The materials have not undergone that fusion in the author's mind which makes a good compilation read like an original work. If they had been submitted to such a process, the resulting book would have been half its present size, and double its present value. As it is, however, Dr. Stewart's treatise will be useful to the diligent student and junior practitioner as a faithful epitome of what is known on the important subjects of which it treats.

